# Explicit and Implicit Feature Contrastive Learning Model for Knowledge Graph Link Prediction

**DOI:** 10.3390/s24227353

**Published:** 2024-11-18

**Authors:** Xu Yuan, Weihe Wang, Buyun Gao, Liang Zhao, Ruixin Ma, Feng Ding

**Affiliations:** 1 School of Software Technology, Dalian University of Technology, Dalian 116024, China; david@dlut.edu.cn (X.Y.); wwh@mail.dlut.edu.cn (W.W.); 32217001@mail.dlut.edu.cn (B.G.); liangzhao@dlut.edu.cn (L.Z.); 2 The Key Laboratory for Ubiquitous Network and Service of Liaoning Province, Dalian 116024, China

**Keywords:** knowledge graph, link prediction, contrastive learning, implicit semantic feature

## Abstract

Knowledge graph link prediction is crucial for constructing triples in knowledge graphs, which aim to infer whether there is a relation between the entities. Recently, graph neural networks and contrastive learning have demonstrated superior performance compared with traditional translation-based models; they successfully extracted common features through explicit linking between entities. However, the implicit associations between entities without a linking relationship are ignored, which impedes the model from capturing distant but semantically rich entities. In addition, directly applying contrastive learning based on random node dropout to link prediction tasks, or limiting it to triplet-level, leads to constrained model performance. To address these challenges, we design an implicit feature extraction module that utilizes the clustering characteristics of latent vector space to find entities with potential associations and enrich entity representations by mining similar semantic features from the conceptual level. Meanwhile, the subgraph mechanism is introduced to preserve the structural information of explicitly connected entities. Implicit semantic features and explicit structural features serve as complementary information to provide high-quality self-supervised signals. Experiments are conducted on three benchmark knowledge graph datasets. The results validate that our model outperforms the state-of-the-art baselines in link prediction tasks.

## 1. Introduction

Knowledge graphs link heterogeneous data from different domains to enhance the integration and encode factual information as triples (of the form head entity, relation, tail entity) [1,2]. Knowledge graphs determine the relations between entities and play an important role in various natural language processing scenarios, such as question answering [3] and recommend systems [4]. But in the real world, knowledge graphs still suffer from an incompleteness problem: some certain crucial links are absent, which results in poor performance on downstream applications [5]. Therefore, the knowledge graph link prediction task, which aims to automatically predict and infer whether there is a relation between the head and tail entities, is crucial for the construction and validation of the triples [6,7]. To accomplish the link prediction task, various models have been proposed [8,9,10]. The traditional methods learn embedding vectors by mapping entities and relations to a low-dimensional space and evaluate the rationality of the triples, including translation-based methods, such as TransE [11] and TransR [12], and tensor decomposition models, such as DistMult [13] and RESCAL [14]. Recently, knowledge graph link prediction has been further enhanced by many graph neural network (GNN) approaches. For example, CompGCN [15] expands the node updates by Graph Convolutional Networks (GCNs) and models multi-relations to explore the topology of the graph. RED-GNN [16] leverages GNNs to progressively propagate a query to conduct knowledge graph reasoning. These methods effectively extract their neighbor information through the topological structure of entities on the knowledge graph. To improve the accuracy of knowledge graph link prediction tasks, recently, contrastive learning frameworks constructed from GNN backbones have been employed. Since they can provide external supervised-signals [17,18], contrastive learning constructs positive and negative pairs from different views [19,20]. Then, by narrowing the embeddings of positive samples while distancing the negative samples, they improve the performance of the model.

**Figure 1:**
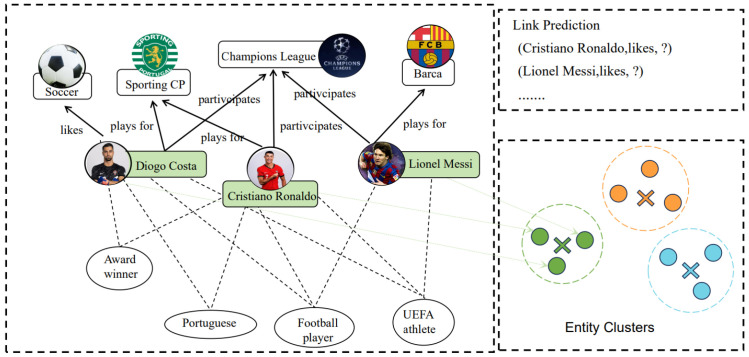
Entity implicit semantic features explanation diagram. It delineates within the embedding space entities clustered together exhibit semantic associations, particularly among those that share identical concepts. This characteristic enriches entity representation. Rectangles represent the entities in the knowledge graph, and ellipses represent the conceptual information of the entities.

Although these methods can successfully capture the local neighborhoods of entities, they still have some limitations: (i) They only focus on explicit links, ignoring the semantic association among entities. For instance, in Figure 1, the entities *Diogo Costa, Cristiano Ronaldo and Lionel Messi* have the same concepts *Person, UEFA athlete and Ball player*, suggesting they might be the same type of athletes. During training, suppose we know the triple (*Diogo Costa, likes, soccer*). Thus, incorporating *Diogo Costa’*s information into *Cristiano Ronaldo* and *Lionel Messi* provides a strong signal for inferring the correct answer *soccer*. Moreover, *Diogo Costa* and *Cristiano Ronaldo* have more shared concepts than *Lionel Messi*, which suggests a stronger connection between them. This allows for the representation of an entity that can also benefit from nodes that are not directly connected but have similar semantics. The exploration of these implicit features can liberate entities from their reliance on neighboring entities and enhance the distinctiveness of entities from a higher-order perspective. (ii) Employing contrastive learning with the random dropping of nodes can result in an incomplete entity’s neighborhood information. Furthermore, self-supervised optimization is confined to the triple level, precluding the acquisition of high-quality self-supervised signals.

To tackle these problems, we propose an effective framework, named as the **E**xplicit and **I**mplicit **F**eature **C**ontrastive **L**earning (**EIFCL**) model for knowledge graph link prediction. The proposed implicit feature encoding module is designed to capture semantic associations of entities. Specifically, by applying clustering algorithms in the embedding space, entities with potential associations are divided into the same cluster. The Jaccard similarity index is used to measure the strength of the semantic association between two entities based on their concept sets. Ultimately, this process encodes the latent semantic features of the entities. Subgraphs are extracted from the neighborhoods of the entities to preserve the explicit linking structure of entities. Instead of performing an aggregation procedure in the general graph, the proposed subgraph mechanisms allow dynamic entity embeddings by only performing message passing in the context subgraph. The explicit structural features of entities are encoded from the most relevant contextual entity vectors. Utilizing these two types of features as supervisory signals for contrastive learning, the model is jointly optimized with a contrastive loss to augment its understanding of the explicit linkage information and implicit semantic features of entities during the pre-training phase. Subsequently, during the fine-tuning phase, the entity representations are updated to better accommodate the link prediction task. Our contributions are as follows:

(1) We develop a novel framework, named EIFCL for the knowledge graph link prediction task, that can effectively exploit both implicit semantic and explicit structural features of each entity, providing high-quality self-supervised signals for contrastive learning through these two complementary information sources.
(2) We explore the potential associations among entities, modeling and encoding their implicit semantic features based on the clustering characteristic in latent space, enabling high-order but informative entities to contribute to our model.
(3) A feature extraction module is designed to preserve local contextual information; the explicit structural feature is encoded through the subgraph mechanism, which enables the dynamic representation of neighbor entities to adapt to various contexts.
(4) We conduct experiments on three benchmark knowledge graph link prediction datasets. The results validate that our model achieves performance superior to those of the state-of-the-art models, including traditional and GNN-based methods for link prediction tasks.

The structure of this article is as follows. Section 2 presents the existing methods in knowledge graph link prediction. Section 3 describes the preliminaries and strategies for building our model. Section 4 shows the performance of EIFCL. The conclusion is drawn in Section 5.

## 2. Related Work

In this section, we initially present traditional methods in knowledge graph link prediction, including translation-based models, tensor decomposition methods and GNN-based models, then delineate the principal challenges they encounter. Following this, we explore the application of contrastive learning within the knowledge graph link prediction.

### 2.1. Link Prediction Task

Knowledge graph link prediction is the task of exploiting the existing facts in a KG to infer missing ones. This amounts to guessing the correct entity that completes <h, r, ?> (tail prediction) or <?, r, t> (head prediction). For the sake of simplicity, when talking about head and tail prediction globally, we call the source entity the known entity in the prediction, and the target entity the one to predict [21,22].

### 2.2. Traditional Methods

**Translation-based models**. Translation-based models treat relations as a translation between head entities and tail entities. The resulting vector is measured by distance functions such as L1 or L2 norms. TransE [11] enforces the tail embedding to be close to the combination of the head and the relation embedding. TransR [12] maps entities and relations to different spaces. Recently, some translation-based models have replaced translation execution with rotational transformation operations. RotatE [23] represents relations as rotations in a complex latent space, which demonstrates that rotation allows one to correctly model numerous relational patterns, such as symmetry/anti-symmetry, inversion and composition. TorusE [24] is motivated by the observation that the regularization used in TransE forces entity embeddings to lie on a hypersphere, thus limiting their capability to satisfy the translational constraint.

However, the optimization at the triple level, without adequate consideration of higher-order structural information, limits the model’s capability to capture complex patterns. There is limited performance on complex relations: translation-based models like TransE are known to perform poorly on 1-to-N, N-to-1 and N-to-N relations. They also struggle with symmetric relations, which can lead to incorrect embeddings for entities and relations involved in such symmetrical relationships.

**Tensor decomposition models**. Tensor decomposition models consider the knowledge graph as a ternary tensor and decompose it into combinations of low-dimensional vectors, namely, embeddings of entities and relations. By considering all relation embeddings as a diagonal matrix, DistMult [13] significantly reduces the parameter space. However, as the scoring function is commutative, this treats all relations as symmetric relations. ComplEx [25] employs the same diagonal constraint as DistMult, but in a complex space: it uses a Hermitian product instead of a bilinear product, and the conjugate transpose is used instead of the usual transpose. ComplEx can successfully model asymmetric relations. Recently, some models combined the head, relation and tail embeddings of composition using formulations different from the strictly bilinear product. TuckER [26] relies on the Tucker decomposition, which factorizes a tensor into a set of vectors and a smaller shared core w. The TuckER model learns w jointly with the KG embeddings. However, it typically involves extensive matrix operations, which may lead to a significant consumption of computational resources. Some tensor decomposition models, due to their nature, can lead to overparameterization. This not only increases the risk of overfitting but also demands more computational resources and longer training times.

**GNN-based methods**. GNN-based methods learn local information of neighbors by a message-passing mechanism. For instance, R-GCN [27] employs an encoder model in GCNs to accumulate relation information. ConvE [28] transplanted a convolutional neural network into a knowledge graph embedding, enabling efficient and straightforward multi-interactions without an excessive number of parameters. CompGCN [15] designs a composition operation that represents all relations using a weighted combination of bases. SE-GNN [29] incorporates multiple semantic evidence levels from neighboring contexts. DRR-GAT [30] obtains dynamic relational representations by analyzing paths between entities in a KG. Unfortunately, the majority of GNN models are constrained to shallow architectures, which limits the contribution of distant but informative nodes to the entity representations.The training complexity of GNNs is often linear with respect to the number of neighboring nodes, which can lead to long training times, especially when dealing with large and dense knowledge graphs.

### 2.3. Contrastive Learning in Knowledge Graph Link Prediction

Contrastive learning, developed in the computer vision domain, leverages the technique of random cropping to generate multiple views of images, serving as positive samples for contrastive learning. Recently, this approach has also been integrated into the knowledge graph link prediction task. Some methodologies focus on the random dropping out nodes to create multiple views [31,32], while others employ triplet-level contrastive learning to extract common features between neighboring entities. KGE-CL [17] takes into account the homogeneity between entities and entity-relation pairs, leveraging four types of patterns of entity-relation pairs to learn more effective representations of the nodes. HeCo [33] proposes to learn information from meta-path and schema views, respectively, optimizing the representations of the nodes using meta-path neighbors. SMiLE [34] introduces a schema and uses it to select specific neighbors of the entity as positive samples while choosing nodes with corresponding identical schema as hard negative samples.

The above methods mine the topological structure of neighbors to generate supervision signals. Unlike them, our approach captures the implicit associations among entities at the semantic level and preserves explicit entity linkages via subgraph encoding, offering enriched and high-quality supervisory signals.

## 3. Methods

**Figure 2:**
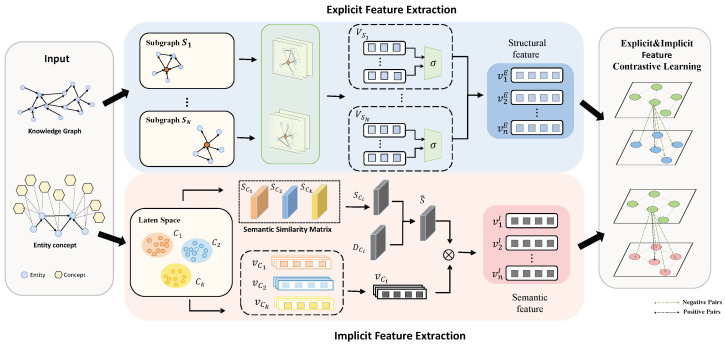
The architecture of our proposed EIFCL model, which has two parts: explicit feature contrastive learning module (**up**) and implicit feature contrastive learning module (**down**).

In this section, we first introduce the preliminaries and notation for our model. Then, the strategies and motivations are described for building explicit and implicit feature contrastive learning in Section 3.2 and Section 3.3, respectively. Finally, the training process is introduced in Section 3.4. The overall architecture of the model is shown in Figure 2.

### 3.1. Notation and Preliminaries

**Notation**. A knowledge graph is a collection of entities and relations, denoted by G=(E,R,T), where E represents the entity set, R represents the relation set and T is the set of all entity concepts. Entities and relations are stored in a knowledge graph in the form of triples (h,r,t), where h,t∈E and r∈R. Each entity e∈E can be associated with one or several concepts, $t_{1},t_{2},\ldots ,t_{n}\in T$.

**The knowledge graph link prediction task**. The objective of the model is to investigate entity representations following the guidance of explicit and implicit feature signals, enabling effective execution of link prediction tasks in the missing links of an incomplete G. Specifically, in the ideal case, the score of the correctly connected triples should be higher than those of corrupted negative ones.

### 3.2. Explicit Structural Feature Extraction

Within a knowledge graph, entities that are explicitly linked through relations often share certain information. Current GNN-based methods for knowledge graph link prediction have demonstrated efficacy due to their ability to capture homophilic characteristics among neighboring entities. However, they propagate messages throughout the entire graph, ignoring the unique structure of each entity. For example, when entity *Apple* is a neighbor entity of *Steve Jobs*, it represents the meaning of a company, but in some contexts, it represents a fruit. This arises because the neighborhood structure varies for each entity, leading to different contexts, and thus the same entity should have a representation that aligns with the context at hand in various situations. Consequently, a subgraph mechanism is introduced to capture the unique structural features of each entity.

**Subgraph mechanism**. Each entity is regarded as a central node. The random walk algorithm is employed to sample local neighbor entities to construct subgraphs for each central entity as a context. For an entity *e*, the subgraph obtained through the sampling is represented as $g_{s}\left(e\right)$. In the subgraph, the set of all neighboring entities set is defined as follows:

(1) $$N_{s}=\left\{e_{i}|e_{i}\in g_{s}\left(e\right),e_{i}\in \right.\left.G\right\}$$

To expedite the training of the model, the Node2vec [35] algorithm is employed to initialize the representations of the entities within each subgraph. The embedding of entities in the subgraph is defined by $V_{s}=\left\{v_{1},v_{2},\ldots ,v_{|N_{s}|}\right\}$, where $|N_{s}|$ represents the number of entities in subgraph $g_{s}$.

**Figure 3:**
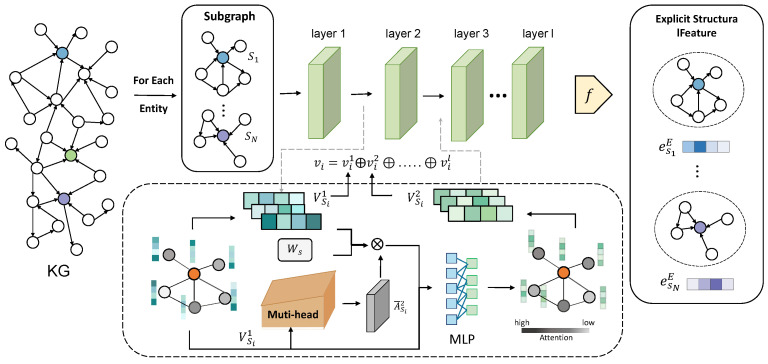
Illustration of our proposed explicit feature encoding. Each layer shifts the embedding of all entities in subgraph $g_{s}$.

To ensure that neighboring entities align with the current context, the message propagation and attention mechanisms operate solely within the confines of individual subgraphs, thereby permitting a neighboring entity to possess distinct vector representations across different subgraphs. See Figure 3.

We initially introduce a multi-head attention mechanism to compute the attention scores of each neighboring entity: given two entities $e_{i}$ and $e_{j}$ in the subgraph $g_{s}$, for each head, we calculate the following:

(2) $${\bar{A}}_{ij}^{l}=\frac{\exp\left({\left(W_{1}v_{i}^{l}\right)}^{T}\left(W_{2}v_{j}^{l}\right)\right)}{\sum_{t=1}^{\left|N_{s}\right|}\exp\left({\left(W_{1}v_{i}^{l}\right)}^{T}\left(W_{2}v_{t}^{l}\right)\right)}$$

where matrixes $W_{1}$ and $W_{2}$ are learnable parameters, and *l* is the number of translation layers stacked in Equation (4).

By performing message passing across all entities in the context subgraph, the entity embedding $V_{s}^{i}$ is updated to be $V_{s}^{l+i}$.

(3) $$V_{s}^{i+1}=MLP\left(W_{s}V_{s}^{i}{\bar{A}}^{i}+V_{s}^{i}\right),i=0,1,\ldots ,l-1,$$

where $W_{s}\in R^{d_{l}\times d_{l+1}}$ is a trainable layer-specific parameter. Due to the fact that the scale of the neighborhood information retained in each layer is different, applying only the output of the last layer $v_{i}^{l}$ is not comprehensive enough. Hence, we retain the sequence of output vectors from each layer $\left\{v_{i}^{\left(1\right)},v_{i}^{\left(2\right)},\ldots ,v_{i}^{\left(3\right)}\right\}$ of entity *v*. By concatenating the embedding from each layer, we aggregate the information from each layer and obtain the representation of the entity as follows:

(4) $$v=v^{0}⊕v^{i}⊕\ldots ⊕v^{l-1}$$

where ⊕ represents the operation of concatenation. Instead of focusing on how to generate a single optimal entity representation, our goal is to allow a neighbor entity to have different representations in different subgraphs. Furthermore, these typical neighboring entity representations are encoded as explicit features for the central entity in the subgraph.

**Explicit feature contrastive learning**. For each central entity, the subgraph mechanism generates a set of representations that fully fit the context based on the different neighborhood structures. The structural feature based on explicit connection relationships between entities is encoded to construct supervised signals for contrastive learning. We use the following function *f* to uniformly aggregate the features of each neighbor:

(5) $$f\left(N_{s}\right)=\sigma \left(\frac{1}{\left|N_{s}\right|}\sum_{e_{n}\in N_{s}}C_{e_{n}}\right)$$

where σ is the activation function and set to tanh, $|N_{s}|$ represents the number of entities in subgraph $g_{s}$ for the center entity *e* and $C_{e_{n}}$ is the representation of the features of the neighbors, which is obtained by a feedforward layer for capturing the interaction between neighbors:

(6) $$C_{c_{n}}=W_{c}\left(v_{e_{n}}\right)+b_{c}$$

where $W_{c}\in R^{d\times d}$, $b_{c}\in R^{d}$ are learnable parameters and d is the dimension of the embedding. The final vector $v^{E}$ containing the unique neighbor structure feature representation of the central entity in subgraphs is defined as follows:

(7) $$v^{E}=f\left(N_{S}\right).$$

Contrastive learning is used to enhance the representation of each entity with its local connections in the subgraph. For entity e, the learned representation $v^{E}$ and *v* form the positive samples, and other embeddings of entities are naturally considered as negative samples. After defining the positive and negative samples, we apply the InfoNCE loss function to conduct contrast estimation:

(8) $$L^{E}=-\log\frac{\exp\left(\phi \left(v_{i},v_{i}^{E}\right)/\tau \right)}{\exp\left(\phi \left(v_{i},v_{i}^{E}\right)/\tau \right)+\sum_{g\in \{S,E\}}\sum_{k\neq i}\exp\left(\phi \left(v_{i}^{g},v_{k}^{g}\right)/\tau \right)}$$

where τ is the adjustable temperature hyperparameter that controls the sensitivity of the score function to balance between uniformity and tolerance, and we utilize cosine similarity as the scoring function ϕ.

### 3.3. Implicit Semantic Feature Extraction

Although existing methods have fully explored the interaction information between entities with explicit linking relationships, there may still be some implicit common features between entities without obvious connections, enriching entity representation and improving the discrimination between entities.

Therefore, the expectation-maximization algorithm [36] algorithm is used to obtain entity clusters in embedding space in order to find entities with potential latent associations, then conceptual information is applied to calculate the degree of semantic association; implicit semantic features of each entity are aggregated from each cluster. Thus, distant but informative nodes can also contribute to entity representation.

**Cluster division**. Inspired by [37], we partition the entities into clusters in the embedding space, aiming to discover entities with potential common features and encourage intra-cluster compactness and inter-cluster separability in the latent space, since the tendency of similar entities is to be proximally distributed within the vector embedding space.

The expectation-maximization clustering algorithm is employed, since it is relatively stable and less prone to becoming stuck in local optima. It can be applied to various probabilistic parameter models with hidden variables and has strong generality. At the same time, its implementation is relatively simple with fewer parameters, reducing the complexity of parameter tuning. It should be acknowledged that there are many clustering algorithms, such as spectral clustering, which map data points to a low-dimensional space defined by the eigenvectors of the Laplacian matrix of the graph. But due to the large amount of matrix computation, it consumes more memory resources and time. At the same time, it requires a uniform number of midpoints in each cluster, which is difficult in the vector space of entities.

The expectation-maximization algorithm is employed to obtain clusters of entities, yielding the entity embedding set $\left\{v_{1},v_{2}\ldots v_{i}\right\}$, having randomly initialized the vectors $\left\{u_{1},u_{2}\ldots u_{k}\right\}$ as the centroids of *K* entity clusters. In the E-step, $\left\{u_{1},u_{2}\ldots u_{k}\right\}$ is fixed and $Q\left(z^{\left(i\right)}\right)$ can be estimated by K-means algorithm over the embedding of all entities. The current nearest centroid z is calculated for each entity:

(9) $$\begin{array}{c} \begin{array}{cc} Q\left(z^{\left(i\right)}\right) & =P\left(z^{\left(i\right)}|v_{j},u_{1},u_{2},\ldots ,u_{k}\right)\\ \; & \propto \left\{\begin{array}{c} 1,{∥v_{j}-u_{z^{\left(i\right)}}∥}_{2}=\min_{k}{∥v-u_{k}∥}_{2}\\ 0,{∥v_{j}-u_{z^{\left(i\right)}}∥}_{2}>\min_{k}{∥v-u_{k}∥}_{2}. \end{array}\right. \end{array} \end{array}$$

If the entity belongs to cluster *i*, then the distribution $P(z^{\left(i\right)}|v_{j})=1$; otherwise, $P(z^{\left(i\right)}|v_{j})=0$ indicates that the entity belongs to other clusters. In the M-step, we find the optimal parameters $\Theta =\{u_{1},u_{2}\ldots \ldots ...u_{k}\}$ for the current entities in the target function as follows:

(10) $$\begin{array}{c} L=-\sum_{e\in E}\sum_{c_{i}\in C}Q_{i}\left(z^{\left(i\right)}\right)\log\frac{P\left(v_{e_{i}},z^{\left(i\right)}|\theta \right)}{Q_{i}\left(z^{\left(i\right)}\right)} \end{array}$$

minimize *L* to obtain *k* centroids of cluster embeddings $\left\{u_{1},u_{2}\ldots u_{k}\right\}$, collect the entities closest to their centroid and constitute *K* clusters $\left\{C_{1},C_{2}\ldots C_{k}\right\}$.

**Semantic association**. The entities connected by relation imply explicit structural associations between them. However, at the conceptual level of entities, those sharing the same concepts also possess implicit common characteristics, which suggest their semantic similarities. Our objective is to capture these implicit associations to provide high-quality self-supervised signals. Firstly, we denote the concept set for entity $e_{i}$ by $T(e_{i})$. $S_{ij}$ denotes the semantic similarity of entity $e_{i}$ and entity $e_{j}$: it is 0 if there is no correlation between these two entities.

(11) $$\begin{array}{cc} S_{i,j} & =\frac{\left|\left\{t|t\in T\left(e_{i}\right)\cap t\in T\left(e_{j}\right)\right\}\right|}{\left|\left\{t|t\in T\left(e_{i}\right)\cup t\in T\left(e_{j}\right)\right\}\right|} \end{array}$$

where the numerator represents the size of the intersection of the two concept sets of the entities, while the denominator represents the cardinality of the union of the concept sets of the two entities.

The Jaccard similarity of entity concepts measures the number of entities in a common concept. Intuitively, if two entities have more identical conceptual attributes, then their semantic similarity will be higher. Therefore, concept intersection and union ratio can fully consider the semantic similarity between two entities and then obtain their semantic association matrix:

(12) $$\begin{array}{c} {\hat{S}}_{ij}=\left\{\begin{array}{cc} S_{ij}, & e_{i}\in C_{k},\mathrm{and}e_{j}\in C_{k},\\ 0, & \;\mathrm{otherwise},\; \end{array}\right. \end{array}$$

where ${\hat{S}}_{ij}$ is a sparsified adjacency matrix. To alleviate the exploding and vanishing gradient problems, the matrix is normalized as follows:

(13) $$\begin{array}{c} \begin{array}{c} \tilde{S}={\left(D\right)}^{-\frac{1}{2}}\hat{S}{\left(D\right)}^{-\frac{1}{2}} \end{array} \end{array}$$

where $D\in R^{N\times N}$ is the diagonal degree matrix of $\tilde{S}$ and $D_{i,i}=\sum_{j}{\hat{S}}_{ij}$. Therefore, the normalized matrix $\tilde{S}$ is finally obtained.

**Implicit feature contrastive learning**. The final implicit semantic feature of entity *e* is as follows:

(14) $$\begin{array}{c} v^{I}=\sum_{e_{i}\in C_{k}}\tilde{S}v_{e_{i}} \end{array}$$

**Figure 4:**
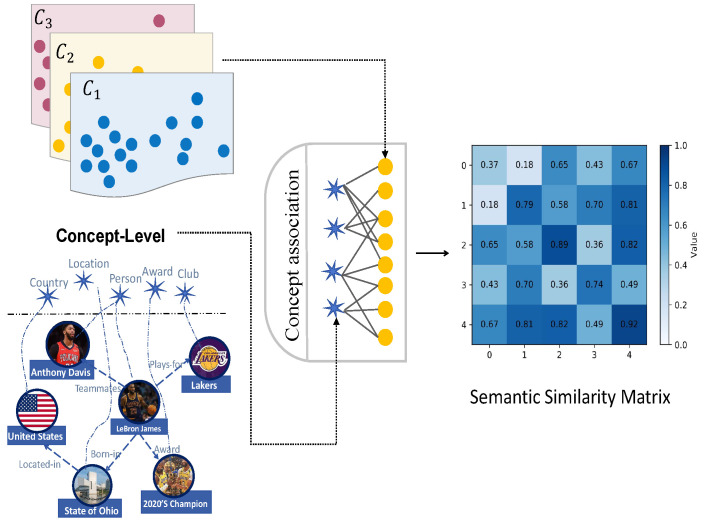
Illustration of the semantic similarity matrix ${\hat{S}}_{ij}$. The similarity matrix is generated by the entity concept association in each entity cluster in latent space.

Then, we feed them into an MLP encoder with one hidden layer, mapping them into the space where the contrastive loss is calculated:

(15) $$\begin{array}{cc} v & =W^{2}\sigma \left(W^{1}v_{e}+b^{1}\right)+b^{2},\\ v^{I} & =W^{2}\sigma \left(W^{1}v_{e}^{I}+b^{1}\right)+b^{2}, \end{array}$$

where σ is the ELU activation function. Both the weight matrix $\left\{W^{1},W^{2}\right\}$ and bias parameters $\left\{b^{1},b^{2}\right\}$ are learnable parameters. Then, we employ contrastive learning to estimate the lower bound of the mutual information between *v* and $v^{I}$. The same entity representation forms positive pairs while the other embeddings naturally serve as negative samples. Therefore, the loss function for exploring the implicit feature in contrastive learning is defined as follows:

(16) $$\begin{array}{c} L^{I}=-\log\frac{e^{\left(\phi \left(v_{i},v_{i}^{I}\right)/\tau \right)}}{e^{\left(\phi \left(v_{i},v_{i}^{I}\right)/\tau \right)}+\sum_{i\neq k}e^{\left(\phi \left(v_{i},v_{k}\right)/\tau \right)}+\sum_{i\neq k}e^{\left(\phi \left(v_{i}^{I},v_{k}^{I}\right)/\tau \right)}}, \end{array}$$

where τ is the adjustable temperature hyperparameter that controls the sensitivity of the score function to balance between uniformity and tolerance, and we employ cosine similarity as the score function ϕ. See Figure 4.

### 3.4. Training Objective

**Prediction Function**. The task of link prediction in a knowledge graph involves predicting the existence of a link between a given triple (h,r,t). Specifically, it aims to assign higher scores to correct triplets compared with incorrect ones, indicating the likelihood of a valid link. Inspired by [15], we propose to construct entity-relation pairs, using the relation-aware embedding of the head entity *e* as $v_{h}^{r}=\Phi \left(v_{e},v_{r}\right)$, where Φ can be subtraction, multiplication, rotation or circular-correlation:

- Subtraction (Sub): $\Phi \left(v_{e},v_{r}\right)=v_{e}-v_{r}$;
- Multiplication (Mult): $\Phi \left(v_{e},v_{r}\right)=v_{s}\cdot v_{r}$;
- Rotation (Rot): $\Phi \left(v_{e},v_{r}\right)=v_{s}\circ v_{r}$;
- Circular-correlation (Corr): $\Phi \left(v_{e},v_{r}\right)=v_{s}★v_{r}$.

We adopt multiplication in our model as the implementation of entity-relation composition. The plausibility of a triple is assessed and quantified using $\sigma (v_{h}^{r}\cdot v_{t})$ as a prediction scoring function.

**Training Process**.To preserve both implicit and explicit features for the entity representation, we optimized the contrastive loss in pre-training and further optimized the model in the fine-tuning stage to accommodate downstream link prediction tasks. In the pre-training, the explicit and implicit feature contrastive learning mentioned in Equations (8) and (16) are employed to optimize the model parameters. The loss function in the pre-training is constructed as follows:

(17) $$L=\sum [\lambda \cdot L^{E}+\left(1-\lambda \right)\cdot L^{I}]$$

where λ represents a balancing coefficient that controls the weights of the explicit feature loss and the implicit feature loss.

After obtaining the model parameters in the pre-training stage, we use fine-tuning to update the entity representations, which is more suitable for link prediction tasks. For a given triple (h,r,t), pollute the tail entity by randomly replacing head and tail entities (h′,r,t′). We use the relation-aware embedding of the head entity *e* as $v_{h}^{r}=\Phi \left(v_{e},v_{r}\right)$, where Φ can be subtraction, multiplication or circular-correlation, etc. We adopt multiplication in our model as the implementation of entity-relation composition. Then, the training objective in the fine-tuning stage is

(18) $$L=\sum log\left(\phi_{r}\left(v_{h}^{r},v_{t}\right)\right)+\sum log\left(\phi_{r}\left(v_{h\prime }^{r},v_{t^{\prime }}\right)\right)$$

where $\phi_{r}\left(v_{h}^{r},v_{t}\right)=\sigma \left(v_{h}^{r}\cdot v_{t}\right)$ is a function to measure the degree of matching between the head entity and tail entity under the relation *r*, and σ denotes the sigmoid function.

### 3.5. The Time Complexity

**Process**. The training process of the model is bifurcated into two stages: pre-training and fine-tuning. During the pre-training phase, the model is expected to learn explicit structural information and implicit semantic information in a self-supervised manner. The parameters are adjusted by minimizing the losses associated with both types of information. In the fine-tuning stage, the model is expected to be better suited for the task of knowledge graph link prediction. This is achieved by constructing negative sample pairs through the random corruption of tail entities, thereby minimizing the loss and enhancing the prediction accuracy through fine-tuning.

**Time Complexity**. Initially, we sample the neighboring entities around each entity to construct a subgraph of the entity. Assuming that obtaining one-hop neighbors of an entity from its neighborhood takes O(1), and sampling N neighboring entities takes O(N), when the number of entities trained per epoch is M, we require O(N*M) for each epoch. Subsequently, in the semantic feature encoding module, a semantic association graph is generated, and an association matrix is computed. Assuming the worst-case scenario where each entity within the same epoch necessitates computation, this requires O(M*M). Therefore, the overall time complexity for graph generation is O(N*M + M*M). The time consumption of experiment on the FB15k, FB15K-237 and HumanWiki datasets are shown in Table 1.

## 4. Experiments

### 4.1. Experimental Settings

**Datasets**. We conduct experiments on FB15k [11], FB15k-237 and HumanWiki [38]. FB15k is a subset of Freebase, and is widely adopted as a benchmark dataset. FB15K-237 is a filtered version of FB15k that includes 237 kinds of relations. The HumanWiki dataset is derived from Wikidata by filtering out all triples that involve a head entity related to the concept of “human”. To incorporate entity concept information for implicit feature contrastive learning, we utilize the dataset developed in [38]. For the link prediction task, an equal number of positive and negative edges are generated. Detailed statistics of these three datasets can be found in Table 2.

**Figure 5:**
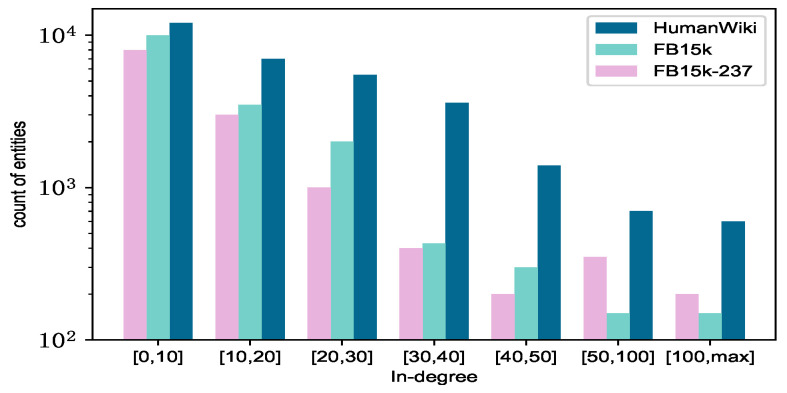
The number of different frequency entities on FB15k, FB15k-237 and HumanWiki benchmark datasets.

To provide a granular presentation of the characteristics of each dataset, we conduct a statistical analysis on the three datasets, FB15k, FB15k-237 and HumanWiki datasets, with some data referencing KRACL. The result is shown in Figure 5.

(i) It can be observed from the figure that FB15k is more sparse compared to FB15k-237. The data in FB15k exhibit a power-law distribution, indicating a significant skew in the information content of the triples within the knowledge graph. Only a small subset of entities with high frequency play a pivotal role in training, while the majority of entities have a minimal impact, leading to a pronounced sparsity in the data. (ii) FB15k-237 is selected from the original FB15k dataset, retaining relationships with at least 50 training triplets. Although the number of relationships has decreased compared to FB15k, the richer number of triplets suggests that more entities may be linked to more neighboring entities. (iii) The HumanWiki dataset is derived from Wikidata by filtering out all triplets involving head entities related to the concept of “human”. Compared to the other two datasets, the distribution is relatively uniform.

**Baselines**. We compare the proposed model with three categories of representative models for link prediction. The first category consists of translation-based models, including TransE, TransR and PairRE. The second category consists of tensor decomposition models, including ComplEx-N3, TypeComplex and SANS. The third category consists of models based on GNNs, including the Node2vec, the multi-relational model CompGCN and the SOTA approach SMiLE. All models are described below:

- TransE [11]: It is the most representative translation-based model that enforces the tail embedding to be close to the combination of the head and the relation embedding.
- TransR [12]: It is a classical translation-based model that defines a relation-specific matrix for each relation and maps entities from the entity space to the relation space.
- ComplEx-N3 [39]: It is a tensor decomposition model that improves the ComplEx model in the aspect of regularization.
- TypeComplex [40]: It is a tensor decomposition model that improves the TDB methods with additional type information.
- SANS [41]: It is a tensor decomposition model based on DistMult with structure-aware negative samples and a self-adversarial approach.
- PairRE [42]: It is a translation-based model that employs paired vector representations to accommodate diverse and complex relations.
- Node2vec [35]: It is a GNN-based method that combines the random walk and word embedding techniques to capture the structural and contextual relationships between nodes.
- CompGCN [15]: It is a GNN-based method which jointly aggregates entity and relation embeddings.
- SMiLE [34]: It is a state-of-the-art GCL method that introduces schema as priors to enable high-quality negative sampling.

**Details of the implementation**. The entity and relation embeddings are initialized by using Node2vec, and the dimension of the entity embedding is set to 128. Temperature τ is initialized to 0.8 and the balancing coefficient to 0.5. The number of layers in Equation (6) is *l* and the multi-head *A* is set to 4. For the batch size of the pre-training, we use 1024 for FB15k, 2048 for FB15k-237 and 1024 for the HumanWiki dataset. For fine-tuning the batch size, we set all to 256 for three datasets. The random walk algorithm is used to generate the subgraphs: the number of entities in each subgraph is set to 12 for FB15k-237 and 6 for FB15k and HumanWiki datasets. The number of entity clusters in the embedding space is initialized to 15. To identify an optimal set of hyperparameters for our model, we performed a grid search utilizing the validation dataset. On the one hand, grid search is more stable because it explores every combination of hyperparameters specified in a grid, ensuring that the best combination is found if it exists within the predefined grid. In contrast, the random search relies on random sampling. Random search may yield varying results with each execution, introducing a degree of uncertainty. On the other hand, grid search guarantees the identification of the global optimum within the search space, provided there is sufficient time and resources. However, the performance of Bayesian optimization may be influenced by the choice of initial parameters and the prior distribution; improper initialization could potentially lead the search process to become trapped at a local optimum. All the experiments were performed using Pytorch 2.5 on a GeForce RTX 4090 GPU. Adam was adopted as the optimizer to train the proposed model.

**Evaluation Protocol**. We evaluated the performance of our model on the link prediction task in knowledge graph, using two commonly used evaluation metrics [43]: (1) micro-F1 scores and (2) AUC-ROC scores.

### 4.2. Experimental Results and Analysis

Table 3 compares the performance of our model with that of the other methods on FB15k, FB15k-237 and HumanWiki. One can make the following observations:

(1) Compared with the others, our model achieves a competitive result. Specifically, the model performs better than the traditional methods (such as ComplEx-N3, PairRE and TransR); we attribute this superior performance to its ability to capture both explicit entity connections and implicit entity associations in the whole knowledge graph, which is more effective in link prediction tasks.
(2) The GNN-based methods outperform the traditional models in most datasets, providing evidence for the effectiveness of exploring graph features. This approach can propagate shared similar features along entity paths. Node2vec performs well on most datasets, indicating that a random walk can learn continuous feature representations of entities in the subgraph, enhancing the effectiveness and robustness of the framework. Surprisingly, CompGCN is worse than the traditional model, probably because CompGCN only models relational connections: the information within a triple is too unitary. Thus, the entity embedding vectors are overlapping and indistinguishable. This also verifies the necessity of considering implicit association information in our model.
(3) For the self-supervised methods, our model outperforms the state-of-the-art SMiLE. Unlike constraining contrastive learning at the instance level in SMiLE, our model uncovers implicit concept-level associations between the entities and enhances the cohesiveness within the clusters of homophilous nodes, as well as the separability between clusters. With this extra information, the generated entity representations are more informative and discriminatory.
(4) Finally, the experimental results indicate that the proposed model consistently performs better than the others. This is brought about by the feature-enriched contrastive learning objectives. In addition, its slightly weaker performance on the HumanWiki dataset is due to an insufficient number of concepts, where each entity has an average of 6 to 7 concepts in the FB15k dataset, but the entities have only 2 to 3 concepts in HumanWiki. This may result in a deviation of entity associations at the concept-level. In addition, more improvement is obtained on the sparse datasets, such as the FB15k dataset. We speculate that mining entity relevance signals to enrich entity representations can more accurately infer missing entities for connected sparse entities. This is also significant in real-world knowledge graph link prediction scenarios.

### 4.3. Ablation Study

Our proposed model leverages two contrastive learning modules to capture self-supervised signals of both explicit and implicit features in a knowledge graph. To elucidate the contributions of the main components of our model, we consider two ablation variants (explicit feature and implicit feature contrastive learning) of our model. The results of this experiment on the FB15k, FB15K-237 and HumanWiki datasets are shown in Table 4. We observe a significant performance improvement in both micro-F1 and AUC-ROC scores when comparing the full framework (third row) to single-component approaches, further certifying that exploring the explicit and implicit features in a knowledge graph makes fruitful contributions to the link prediction task. Also, the performance degrades when any component is dropped, indicating the effectiveness of the dropped component.

We can observe that, for FB15-237, the contribution of implicit features is more significant than that of the rest of the model. This can be attributed to the fact that FB15-237 is derived by selecting 237 relations from FB15k, resulting in a lower number of relations and a higher degree of entities. As a result, the filtered graph is denser, allowing more abundant local information to be captured. In such cases, implicit features, as an alternative perspective to enrich entity representations, can significantly enhance knowledge graph embeddings.

**Figure 6:**
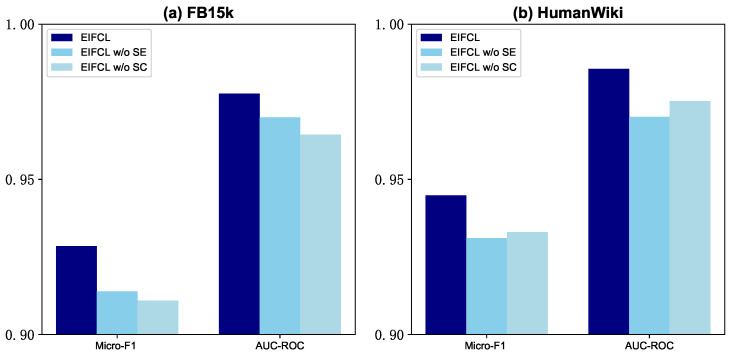
The ablation results of two modules.

To systematically evaluate the contribution of each module in GLSE, we conduct a series of ablation studies on the Subgraph Encoding (w/o SE) and Semantic Clustering (w/o SC). The results are shown in Figure 6. After each module is removed, the model effect decreases to different degrees, which proves the validity of our design choice.

The ablation results of the two modules are analyzed in detail. (i) w/o SE: After the removal of the Subgraph Encoding, we observed a performance decline across both datasets, which indicates the effectiveness of context information. Notably, performance degrades significantly on the dense HumanWiki after the removal of the SE, suggesting that Subgraph Encoding plays a crucial role in making the most of neighbor information. (ii) w/o SC: The results clearly demonstrate that modeling local context with SC is effective on both datasets. Interestingly, the decrease was more pronounced on the sparse FB15K, suggesting that sparse entities rely more on additional semantic information than on local context.

### 4.4. Parameter Sensitivity

**Figure 7:**
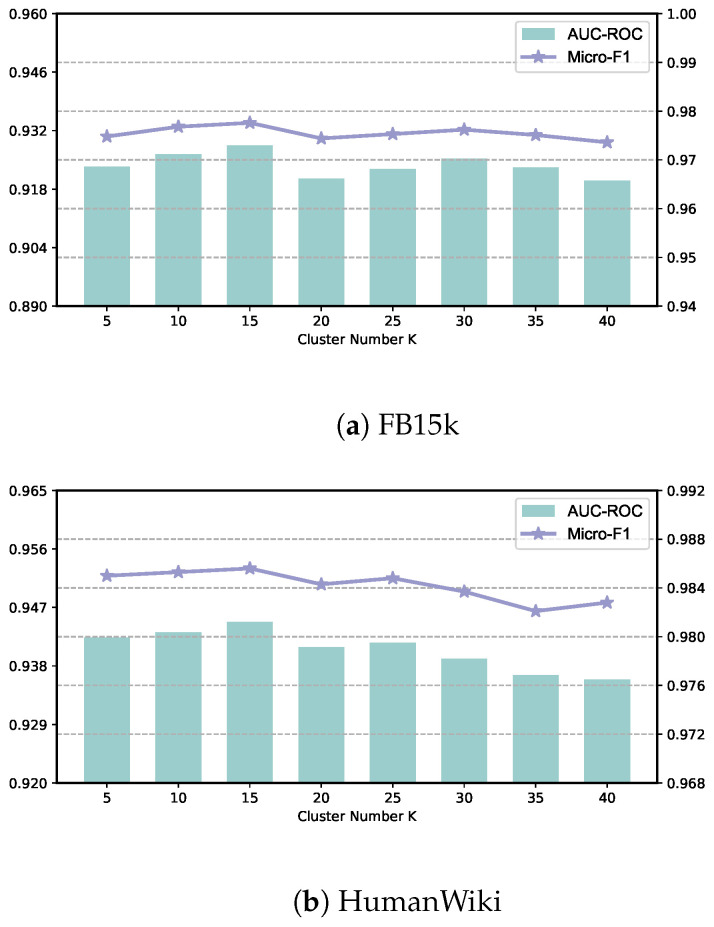
Parameter sensitivity analysis for the cluster number K on FB15k and HumanWiki.

**Impact of the number of clusters** *K*. To examine the impact of *K*, the number of entity clusters, we conducted experiments with different values of *K*. Figure 7 illustrates the performance with varying numbers of clusters (ranging from 5 to 40). The results indicate that the model achieves the best performance when *K* is set to 15. Initially, increasing the number of clusters improves the performance when *K* is less than 15. However, When *K* exceeds 15, the performance worsens by varying amounts. This can be attributed to excessive noise introduced by too many entity clusters or insufficient exploration of potential semantic relationships when the number of clusters is too small. When we set *K* to approach infinity, the performance worsens, indicating the usefulness of exploring implicit features for enhancing link prediction performance. Nevertheless, throughout the process of adjusting *K*, our model consistently outperforms the other approaches, demonstrating its robustness to the number of clusters.

**Figure 8:**
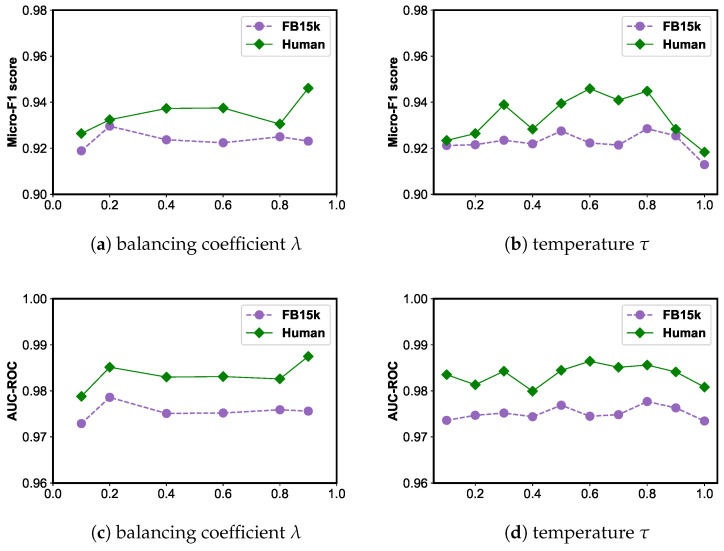
Parameter sensitivity analysis for the balancing coefficient λ and temperature τ on FB15k and HumanWiki.

**Impact of balancing coefficient λ**. In Figure 8a,c, we display how the balancing coefficient λ affects the performance of the model on the FB15k and HumanWiki datasets. The parameter λ balances the proportion of the two kinds of losses in the pre-training of the contrastive learning losses. We choose the values of the parameter λ from $\left\{0.1,0.2,0.4,0.6,0.8,0.9\right\}$ to study its influence. From the results shown, the following observations can be summarized: (1) The worst performance usually occurs for λ=0.2 and λ=0.8, emphasizing the importance of single contrastive loss. (2) The performance of the scenario is better near the central axis, but poor near both ends. This shows that in a balanced state, the model can be reasonably optimized and performs better.

**Impact of temperature τ**. Temperature τ, which is defined in Equations (8) and (16), plays a crucial role in adjusting the attention given to challenging samples in contrastive learning. A higher temperature treats all negative samples equally instead of excessively focusing on more difficult ones. Conversely, a lower temperature coefficient emphasizes challenging negative samples that have a high similarity to the anchor sample, providing a larger gradient to separate them from positive samples. When the temperature coefficient exceeds an appropriate value, the feature distribution of the samples becomes more uniform, leading to stable model performance generated by this loss. To analyze the impact of the temperature parameter τ on our model, we varied τ within the range of 0 to 1. As depicted in Figure 8, as τ approaches infinity, all negative samples are treated equally, leading to the loss of focus on challenging negative samples. Therefore, we selected τ=0.8 to achieve the best results for our model.

**Figure 9:**
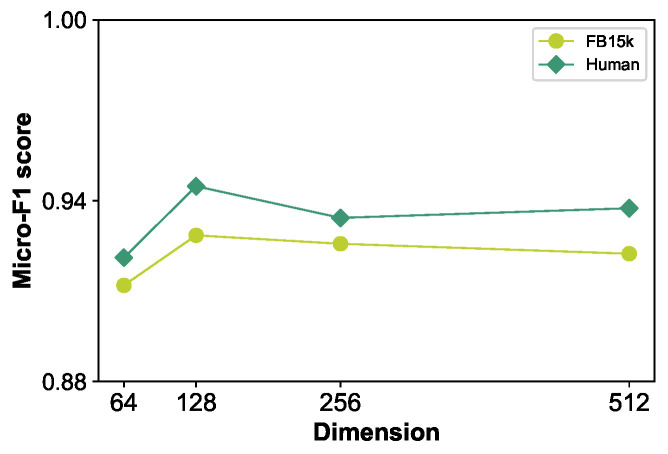
Parameter sensitivity analysis for the dimension d on FB15k and HumanWiki.

**Impact of dimension** *d*. The selection of embedding dimensions typically necessitates evaluation through specific tasks, such as node classification and link prediction, each of which has dimensions that are most suitable. The range of dimensions for entity embeddings spans from tens to thousands, with an optimal dimension often found in the middle. An insufficiently low dimension may lead to inadequate representational capacity of the model, while an excessively high dimension is prone to overfitting. As depicted in Figure 9, as *d* approaches 64, it leads to the loss of focus on insufficient expressiveness of the model, and the choice of embedding dimension k is not necessarily better. In addition to overfitting, it also reduces the efficiency of the model. Therefore, we selected d=128 to achieve the best results for our model.

**Impact of parameters in subgraph**. To explore explicit features, the subgraph is designed to apply aggregation operations exclusively within each subgraph, and is expected to generate entity representations that best fit the context within each subgraph. In this module, we introduce three parameters: the number of entities in the subgraph *n*, the number of translation layers *l* and the number of multi-head attention heads *a*. Table 5 shows the performance on the FB15K and HumanWiki datasets.

In this module, *n* represents the number of sampled entities for a center entity in the subgraph by the random walk algorithm. To explore the impact of different numbers of entities in the subgraph, we selected the number from $\left\{6,8,10,12\right\}$ as the context for the central entities within the subgraph and tested the effectiveness when incorporating them with contrastive learning. We observed that the performance of all three variants is similar to or better than that of the standard approaches, further indicating the effectiveness of the proposed explicit feature exploration and contrastive strategy. Specifically, the results showed that the variant with $|N_{s}|=6$ achieved the best performance among these variants. Surprisingly, an abundance of sampled neighboring entities resulted in a decline in performance, as excessive neighbors not only fail to provide efficient information but also introduce noise, thereby impeding the effective representation of entities.

To examine the influence of the number of layers and attention heads, we explored the performance of the proposed method as these varied. Remarkably, we observed a significantly worse performance when stacking more layers, due to the features of entities within a subgraph becoming similar during the iterative aggregation. Consequently, explicit features fail to provide high-quality supervision signals. Using four layers of contextual translation yielded the best performance across all the datasets, indicating that four contextual translation layers are sufficient to capture complex high-order features in a knowledge graph. Based on these analyses, we set the default values for the number of attention heads and layers to be four.

### 4.5. Analysis of Sparse Entities

**Figure 10:**
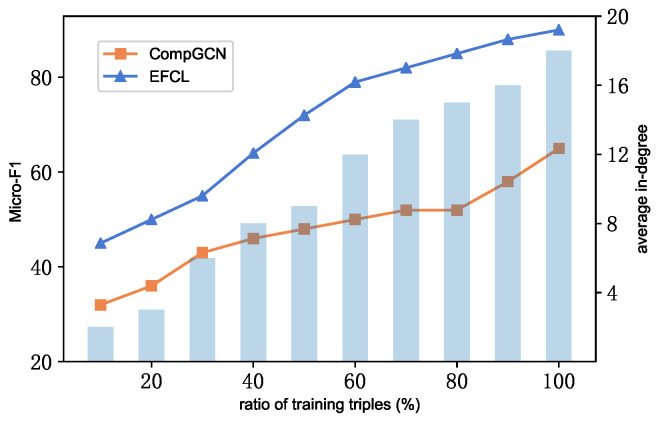
Performance of EIFCL and baseline model on low-degree knowledge graphs.

To verify the effectiveness of EIFCL in sparse entities, particularly the Implicit Semantic Feature Extraction, we perform experiments on entities with different degrees and compare the results with those obtained using CompGCN. For the experimental setup, a random selection of triples is removed from FB15k-237’s training dataset to obtain entities with different degrees. It is important to note that the overall connectivity of the KG is maintained during this process. The results of these experiments are shown in Figure 10.

We observe that all models generally exhibit a decline in performance as the proportion of low-degree entities increases. However, our method demonstrates stable performance without significant decline and consistently surpasses the competitive model in effectiveness. Additionally, with an increasing number of low-degree entities, the performance gap between our method and the baseline model grows more evident. Overall, these findings affirm our approach’s effectiveness, utilizing semantics, in handling sparse entities.

### 4.6. Visualization

To verify the effectiveness of implicit features in the model, or to verify whether it can capture semantically similar entities to improve the accuracy of link prediction, we simulated the entity distribution in vector space using the t-SNE method. In order to effectively observe the accuracy of entity link prediction, we randomly selected six tail entities from the FB15k dataset. For each tail entity, we randomly sampled some head entities connected to it through relationships. Ideally, head entities belonging to the same tail entity should be close because they have similar semantics. Head entities that do not belong to the same tail entity should have clear boundaries, as this will help improve the accuracy of link prediction tasks but also reflect semantic differences.

**Figure 11:**
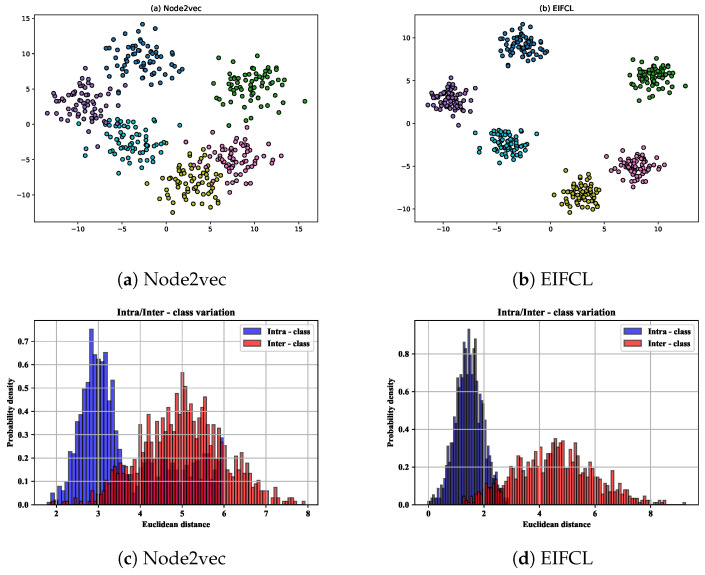
The visualization of entity embeddings on FB15k dataset using t-SNE. Points in same color indicate that they are head entities connected to the same tail entity via a relation.

We visualize these entity embeddings computed with Node2vec and EIFCL, respectively. As shown in Figure 11, model Node2vec in Figure (a) cannot separate entities in different contexts distinctly, especially since there are some overlaps between entities in context Warner Bros. and those in context London. Conversely, entities in different contexts are well separated by utilizing semantics in Figure (b). Moreover, the distance of entities within the same semantic is much closer, while the distribution of different contexts is much wider. Less overlap among clusters demonstrates that the proposed EIFCL effectively models the implicit semantic information of entities while it distinguishes entities of different types from one other.

We perform data statistics on the model separately, specifically using Euclidean distance to represent the distance between data points $d\left(A,B\right)=\sqrt{\sum_{i=1}^{N}{\left(x_{iA}-x_{iB}\right)}^{2}}$. The results are shown in the Figure 11. Comparing (c) with (d), it is found that EIFCL exhibits the distance between data points within a class distributed in smaller areas in the graph, indicating that the tail entities connected to the same entity are closer to each other, which suggests that the clustering within the class is tighter. In contrast, the inter-class distance shows a wider distribution, with data points distributed within a larger Euclidean distance range. This distribution indicates a higher degree of separation between these classes, demonstrating the discriminative power between them.

### 4.7. Large Language Models (LLMs) Comparison and Analysis

Large language models (LLMs) have made significant progress in the field of natural language processing (NLP). In this section, we first provided an overview of LLM models, including Flan-T5, Llama 2 and state-of-the-art Gemma. Then, we quantitatively compare and analyze these LLM models with our model EIFCL on the micro-F1 and AUC-ROC metrics in FB15k-237.

(i) Flan-T5: Flan-T5, developed by Google, represents a significant advancement in the field of large language models (LLMs) designed for natural language processing (NLP) tasks. In terms of architectural specifics, Flan-T5 employs the standard Transformer model with several critical enhancements. It utilizes RMSNorm for pre-normalization, SwiGLU as the activation function and Rotary Position Embeddings (RoPE) for encoding sequence positions. (ii) Llama 2: Llama 2 is a series of large language models (LLMs) released by Meta AI in December 2023, utilizing most of Llama 1’s pre-training settings and model architecture. They use the Transformer architecture, apply RMSNorm for pre-normalization and use the SwiGLU activation function and rotation position encoding. (iii) Gemma: Gemma, developed collaboratively by Google DeepMind and other Google AI teams, represents a significant advancement in the domain of large language models (LLMs) designed for natural language processing (NLP) tasks. Utilizing the same research and technology that underpins the Gemini model, Gemma employs sequence models, Transformer architecture and neural network-based deep learning methods, trained on a large-scale distributed system.

**Figure 12:**
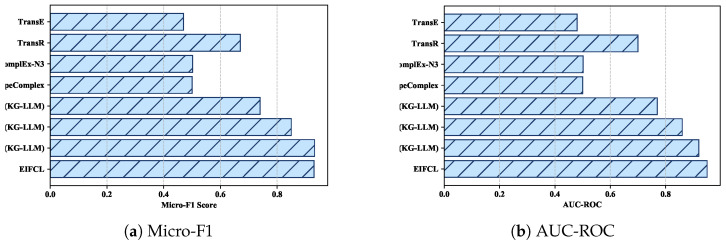
Knowledge graph link prediction performance of our method and large language models (LLMs) on FB15k-237.

The following conclusions can be drawn from the Figure 12. Firstly, it should be acknowledged that all KG-LLM frameworks have achieved more competitive results than traditional models, such as translation-based (TransE, TransR) and tensor decomposition-based models (ComplEx-N3, TypeComplex). The enhanced performance can be ascribed to the knowledge prompts inherent within the KG-LLM framework. These prompts enable the LLM to leverage the relationships network of entities and their interconnections within the knowledge graph. Furthermore, these LLMs already possess basic common sense knowledge from pre-training. As all nodes and relationships are transformed into textual form, this inherent common sense augments their comprehension of the relationships and nodes, thereby augmenting the precision of link prediction. Instruction fine-tuning (IFT) also facilitates this enhancement by compelling the model to focus on a limited set of options.

Secondly, our model has achieved superior performance compared to Flan-T5 and Llama 2, which we attribute to its ability to capture the structural information inherent in knowledge graphs. The text representations favored by large language models (LLMs) are not inherently compatible with graph structural representations: these models are typically trained on vast amounts of textual data, excelling in the sequential processing of linear text information. Textual data, by their nature, follow a linear progression, allowing models to process them sequentially. In contrast, knowledge graphs represent a graph-structured data paradigm where entities and relationships form a complex network. The disparity between the linear structure of text and the graph structure of knowledge is substantial. Converting graph structural information into a format that large models can interpret as text may result in the loss of critical graph structural information, such as the connectivity between entities and the degree of nodes. This loss can prevent the large models from accurately comprehending and leveraging the graph structure.

Lastly, we observed that our model achieved results similar to Gemma’s, which may be because the architecture of Gemma exhibits several distinctive features, including the utilization of Rotary Position Embedding (RoPE) for encoding positional information, and the adoption of an approximate GeGLU non-linear activation function. These elements are instrumental in enhancing the model’s capability to capture long-range dependencies and complex relations.

## 5. Conclusions and Future Work

In this paper, we have proposed a novel framework to mine and encode the complementary information for the knowledge graph link prediction task. We first identified the key challenges of the existing methods, which are ignoring implicit associations in entity semantics and lacking high-quality supervision signals for contrastive learning. Then, we proposed an extraction model to mine the implicit associations through clustering entities in latent space and capturing semantic features from the concept level. Additionally, the contextual structural feature is also encoded and serves as an explicit feature. Furthermore, contrastive learning is introduced in pre-training to fully exploit the complementary information of these two kinds of features and train them in coordination, then the representations of entities and relations are fine-tuned to learn subtler knowledge for the link prediction task. Thus, the model can supplement rich semantic features by implicitly associating entities, while explicit features preserve the neighborhood information of entities. Joint optimization of this complementary information avoids the contextual impairment of contrastive learning using random node cuts. Empirical experiments on three benchmark datasets have demonstrated that our proposed model effectively learns a representation of an entity under the guidance of complementary features. However, this model also relies on sufficient and accurate concepts of entities, which may be inadequate in some knowledge graphs. In future work, we will aim to explore more generalizable sources explained as follows. (i) Utilizing pre-trained embeddings: Pre-trained embeddings from large text corpora can directly supplement missing concepts in knowledge graphs. These embeddings can provide a rich semantic representation that may not be fully captured within the knowledge graph. By aligning or integrating these pre-trained embeddings with the entities, they can enhance the concept information available for link prediction tasks. (ii) Integration of entity prototypes and common-sense information: The supplementation of missing concepts within knowledge graphs can be achieved by leveraging entity prototypes or common sense. For instance, employing Formal Concept Analysis (FCA), excavate deterministic knowledge from knowledge graphs and transform it into concept lattices, providing additional information for link prediction tasks.

## Figures and Tables

**Table 1:** The time consumption (in seconds) of experiment on the FB15k, FB15K-237 and HumanWiki datasets.

Component	FB15k (s)	FB15k-237 (s)	HumanWiki (s)
Total pre-training time	11,972.88	13,077	8945.52
Total fine-tuning time	16,629	18,681.6	12,779.32
Evaluation time	49.88	42.22	44.28

**Table 2:** Dataset statistics.

Dataset	#Entities	#Relations	#Concepts	#Edges	#Triples
FB15k-237	14,541	237	583	248,611	310,116
FB15k	14,579	1208	588	117,580	154,916
HumanWiki	38,949	221	388	105,688	108,199

**Table 3:** Knowledge graph link prediction performance of our method and some baseline models on FB15k, FB15k-237 and HumanWiki datasets. The best results are in bold.

Model	FB15k		FB15k-237		HumanWiki	
	Micro-F1	AUC-ROC	Micro-F1	AUC-ROC	Micro-F1	AUC-ROC
TransE	0.5036	0.5013	0.4778	0.4818	0.4906	0.4931
TransR	0.7196	0.7696	0.6719	0.7076	0.6156	0.6654
ComplEx-N3	0.4963	0.4963	0.5019	0.5034	0.5453	0.5286
TypeComplex	0.8809	0.9390	0.5005	0.5025	0.8017	0.8558
SANS	0.8897	0.9459	0.5003	0.5026	0.7818	0.8369
PairRE	0.8827	0.9267	0.4962	0.4930	0.8007	0.8768
Node2vec	0.8023	0.8891	0.8369	0.8977	0.8013	0.8754
CompGCN	0.6035	0.6359	0.6539	0.7201	0.5688	0.4009
SMiLE	0.9076	0.9653	0.8875	0.9492	0.9340	0.9792
**EIFCL (ours)**	**0.9285**	**0.9776**	**0.9023**	**0.9620**	**0.9448**	**0.9856**

**Table 4:** Ablation study results on FB15k, FB15k-237 and HumanWiki datasets. The best results are in bold.

Model		FB15k		FB15k-237		HumanWiki	
Implicit	Explicit	Micro-F1	AUC-ROC	Micro-F1	AUC-ROC	Micro-F1	AUC-ROC
✓		0.9139	0.9650	0.9010	**0.9623**	0.9311	0.9701
	✓	0.9113	0.9644	0.8863	0.9505	0.9330	0.9752
✓	✓	**0.9285**	**0.9776**	**0.9023**	0.9620	**0.9448**	**0.9856**

**Table 5:** Impact of parameters in subgraph.

	FB15k		HumanWiki	
	Micro-F1	AUC-ROC	Micro-F1	AUC-ROC
l = 2	0.9250	0.9737	0.9814	0.9287
l = 4	**0.9285**	**0.9776**	**0.9856**	**0.9448**
l = 6	0.8849	0.9507	0.8896	0.9571
a = 4	**0.9285**	**0.9776**	**0.9856**	**0.9448**
a = 8	0.9211	0.9742	0.9853	0.9405
a = 12	0.9241	0.9750	0.9831	0.9385
a = 16	0.9216	0.9744	0.9817	0.9319
$|N_{s}|$ = 6	**0.9285**	**0.9776**	**0.9856**	**0.9448**
$|N_{s}|$ = 8	0.9203	0.9743	0.9836	0.9387
$|N_{s}|$ = 10	0.9231	0.9743	0.9863	0.9399
$|N_{s}|$ = 12	0.9254	0.9763	0.9813	0.9339

## Data Availability

Data are contained within the article.
